# Perceived Teacher Discrimination and Depressive Feelings in Adolescents: The Role of National, Regional, and Heritage Identities in Flemish Schools

**DOI:** 10.1007/s10964-022-01665-7

**Published:** 2022-08-20

**Authors:** Charlotte Maene, Fanny D’hondt, Caspar J. Van Lissa, Jochem Thijs, Peter A. J. Stevens

**Affiliations:** 1 Department of Sociology, Ghent University, Ghent, Belgium; 2 Department of Methodology and Statistics, Tilburg University, Tilburg, The Netherlands; 3 Department of Interdisciplinary Social Science, Utrecht University, Utrecht, The Netherlands

**Keywords:** Identity multiplicity, Mental health, Depression, Ethnic/racial discrimination, Secondary education

## Abstract

Adolescents’ identities are multiple, yet there is very little research that investigates the importance of intersecting identities, especially in relationship to teacher ethnic/racial discrimination and mental health. Multiplicity is often approached bi-dimensional (heritage and national identities) yet this study highlights the importance of regional identity. Regions are distinct socio-political contexts in relation to migration and integration dynamics. Hence, this study investigates for different combinations of national, heritage and regional identities (i.e. Flemish, Belgian and Turkish or Moroccan) the relationship between students’ experiences with teacher ethnic/racial discrimination and students’ depressive feelings. Latent Class Analysis of survey data involving a sample of 439 adolescents (*M*_*age*_ = 18, *SD* = 0.93; Girls = 49%) with Turkish (41%) or Moroccan origin in Flanders, shows three identification classes: full integration (35%), national integration (40%) and (weak) separation (24%). All these identity profiles had in common that heritage identification was high, yet they were highly distinct due to variation in national and regional identification. Additional, multilevel modelling showed that nationally integrated adolescents were less depressed than fully integrated adolescents. This finding illustrates the importance of adolescents’ identity multiplicity for understanding their resilience in relation to teacher discrimination.

## Introduction

Research has shown that ethnic/racial discrimination is related with more depressive feelings in adolescents (e.g. Cheon et al., 2020). Yet, research on teacher discrimination has mainly focused on academic outcomes and much less on its psychological consequences (Verkuyten et al., 2019). The present study sought to address this knowledge gap by examining the association between teacher discrimination and depressive feelings. Many studies remain focused on adolescents ethnic or racial identification as a potential protective factor for discrimination (for an overview see Stevens & Thijs, 2018) despite the fact that one decade ago scholars were proposing more research into adolescents’ multiple social identification (i.e. their simultaneous belonging to various important social groups) as a source of resilience (see Baysu et al., 2011). This is typically studied from a bi-dimensional perspective based on the acculturation framework (Berry, 1997) that describes the ways in which individuals combine belonging to an ethnic or racial heritage and (host) national group with each other (e.g. D’hondt et al., 2017).

This study highlights the role of regional/subnational identification in addition to adolescents’ national and heritage identification. Regional identification is relevant in many European societies, as can be witnessed from political parties that are built around sub nationalism/regionalism or research that focuses on regional identification and prejudice towards other regions within a country. Such research on the importance of regionalism has been conducted in Spain (e.g. Catalonia; see Coller, 2002), Belgium (e.g. Flanders, Wallonia; see Niessen et al., 2018), Austria (e.g. Tyrol and Salzburg; see Barth-Scalmani, Kuprian, Mazalh-Wallnig, 2017), Finland (i.e. Purkarthofer; see Humer & Mattila, 2021), and the UK (e.g. Scotland; see Morisi, 2018). This study posits that a three-dimensional approach of social identification in terms of heritage, national and regional identity is relevant for studying adolescents’ resilience with ethnic/racial discrimination and depressive feelings.

### Perceived Teachers’ Discrimination and Depressive Feelings

Ethnic/racial discrimination occurs when people treat others differently—in a more disadvantageous way—based on their ethnicity or race (Verkuyten et al., 2019). Discrimination by teachers can occur or be perceived in various ways, such as in a linguistic way (offenses, verbal abuse), but also instrumentally through biased judgment, lower involvement in mentorship (Jelsma et al., 2022). This type of discrimination can be a severe stressor for students due to a double power imbalance. First, students expect their teachers to be responsible and just. Teachers who make their students feel discriminated are violating their role as moral role models (Stevens et al., 2016). Second, the majority of teachers belong to the dominant ethnic/racial group in society; this is not only the case in Belgium (Agirdag et al., 2016) but also in other countries like the United States, Germany (e.g. D’Amico et al., 2017) or the Netherlands (e.g. Thijs et al., 2019). As such, teachers typically represent the society’s dominant culture. Empirical research has shown that students who feel discriminated by their teacher have lower school belonging (D’hondt et al., 2015) and higher academic futility (D’hondt et al., 2016). Recent research also found a link between teacher discrimination and poorer mental health such as more depressive feelings, anger and suicidal ideation (Jelsma et al., 2022). Thus, it can be expected that ethnic/racial teacher discrimination will be associated with higher depressive feelings for some students.

### Multiple Identification and Resilience

Not all adolescents are equally affected by ethnic/racial discrimination. A resilience perspective makes the distinction between promotive and protective factors (Zimmerman, 2013). Promotive factors are generally good for individuals well-being, irrespective of the absence/presence of stressors, while protective factors reduce the negative impact of stressors such as discrimination. In this field of research, adolescents’ ethnic racial identification has been proposed as an important protective factor (Zimmerman, 2013). Ethnic racial identification involves the degree to which adolescents experience belonging to their ethnic or racial groups (see Ashmore et al., 2004). Belonging is similar to the attachment and closeness to the ethnic/racial group (Ashmore et al., 2004). In the European context, adolescents’ ethnic or racial identity is very much connected to adolescents’ (parents’) nationality-of-origin. Because children typically inherit the ethnic and racial identities of their parents, the term heritage identification is used here.

Ample research has examined the protective role of heritage identification (for overviews see Umaña-Taylor, 2016 and Stevens & Thijs, 2018) but yielded inconsistent results. One reason for this inconsistency might be adolescents’ heritage identification intersecting with other relevant social identities such as national and regional identities. Something that has been overlooked by many studies in the field is that adolescents with a migration background develop cultural and social bonds with various groups (Maes et al., 2014). These adolescents have been submerged into their “new” society’s national culture through education and have been enculturating into their heritage culture through family ties. Their heritage culture is something that sets them apart from adolescents without a migration background but their (sub)national group membership (via their citizenship) is something they have in common with those peers (Dovidio et al., 2007).

To understand multiple identification, Berry’s acculturation framework (1997) provides a solid base. It constitutes of two axes i.e. the extent to which an individual is oriented towards a nationality of origin (“heritage”) and national belonging or the extent to which an individual is oriented towards a (host/new) nationality. The combination of these axes leads to four strategies: (1) Adolescents in the integration strategy are involved both in their heritage and national identity, (2) while adolescents following an assimilated acculturation strategy mainly belong to their national identity and less with their heritage identity. (3) Adolescents who are only involved in their heritage identity follow a separation acculturation strategy, while (4) adolescents who do not belong to either one of these identity groups are marginalized. Although the acculturation framework is bi-dimensional, it has been used successfully in other studies as an important theoretical footing to study multiple identification (e.g. Maehler et al., 2019) because it allows to hypothesize how people position themselves towards potentially multiple in-groups.

#### Integration and discrimination

The notion that integration is the most promotive and beneficial—especially in the absence of discrimination—has been put forward by different scholars (Schotte et al., 2018) and the effects of discrimination for integrated adolescents remain understudied (Nguyen & Benet-Martínez, 2013). The few studies that have focused on this issue suggest that integration is a non-adaptive identification strategy when discrimination occurs presumably because the dual identification is rejected or deemed incompatible (Berry & Hou, 2017).

#### Assimilation and discrimination

In general, adolescents with an assimilated identity tend to be in an intermediate to moderately positive position with regard to their well-being (Berry & Hou, 2017): they tend to be less adjusted than adolescents with an integrated identification but more adjusted than marginalized students. There is an indication that assimilation might be the most adaptive acculturation strategy when adolescents feel ethnically discriminated because the discrimination applies to a group that they do not strongly belong to, namely their cultural heritage group (Berry & Hou, 2017).

#### Separation and discrimination

Overall, separation is associated with moderate negative developmental health outcomes for minorities such as depression (Ünlu Ince et al., 2014) and anxiety disorders (Bulut & Gayman, 2020). In the presence of ethnic/racial discrimination or threat, separation functions as a protective factor (Baysu et al., 2011). Another study showed that separated individuals report low levels of discrimination and depression (Cheon et al., 2020). Both studies have in common that separated individuals do not seem to be maladjusted when ethnic/racial discrimination is taken into account and although the underlying mechanisms for this protective role remains understudied (See also Neblett et al., 2012), one possible explanation is that these individuals ascribe discrimination to an outgroup (i.e. a group to which they do not belong) and therefore their self-concept remains undamaged (Neblett et al., 2012).

#### Marginalization and discrimination

Empirical descriptions of marginalized individuals (with weak heritage and national identification) are limited. One study found that marginalization seemed to be associated with depressive feelings and low levels of social support (Bulut & Gayman, 2020). Adolescents’ experiences with discrimination were not taken into account in that study. Some empirical evidence suggests that marginalization is the least adaptive to buffer the negative effects of discrimination on mental health (Cheon et al., 2020, Berry & Hou, 2017). Discrimination might be particularly harmful for marginalized adolescents because they are socially isolated from important ethnic/racial groups and therefore lack a support network to cope with discrimination (Klein et al., 2020; Nguyen et al., 2017).

### The Role of Regionalism

The process of European unification has led to regionalism as a response to a distant (national or international) structure of authority (Mazzoleni & Ruzza, 2018). Regional identity realizes itself through different societal actors such as politicians, intellectuals (Dehdari, & Gehring, 2022) but also popular culture and sports (Tsai, 2021). As a consequence, inhabitants of these regions can develop dual loyalties (Maene et al., 2021). Few studies have looked into this difference for inhabitants with a migration background. Evidence suggests that the combination of regional and national identification is not always self-evident for inhabitants with a migration background and that internal variation occurs (Clycq et al., 2021). This can potentially be explained by two different logics. One logic posits that people with a migration background adapt to the political and cultural norms of their direct environment—especially when they are socialized by state institutions such as secondary education for adolescents—and therefore the specific built up of one’s regional/national identification will mirror the majority’s configuration (Bilodeau et al., 2010). Another logic however is that regional identity politics often use an ethnic distinctiveness rhetoric to distinguish regionals from nationals and (descendants of) immigrants as they are (perceived to be) culturally different (Fagerholm, 2016). In this scenario, national identification rather than regional identification is easier for people with a migration background as it is a national civic discourse that ensures inclusion and not the discourse in the direct (regional) environment itself (Bilodeau et al., 2010). Because of both possibilities, it is necessary to investigate this empirically.

### The Flemish Context

Belgium is a federal state within the European Union and its sub nations are responsible for the organization of cultural and socio-economic life. The focus of this study is on Flanders as one of those sub nations. The Belgian nation state and the Flemish region developed a different stance towards cultural diversity and migration. The former highlights citizenship through civic and formal norms (e.g. voting rights, economical participation etc.), while the latter stresses maintenance of cultural norms and an overall more negative stance towards immigration (Van Praag et al., 2019). This discourse results in an overall low identification with Flanders among ethnic minorities adolescents (Clycq et al., 2021)—yet some adolescents do feel connected to Flanders and express a considerable identity struggle because of the assimilative, Flemish educational policy towards cultural diversity (Driezen et al., 2021). This suggests that adolescents who opt for a dual identification (in terms of their regional and national identity) may be at risk for lower well-being.

In this study this focus is on high school students’ from Turkish and Moroccan background. Belgium has a long history of immigration from these nations, tracing back to labor migration during the post-Second World War period of rebuilding the industrial economy, and continuing through family reunification (Vanduynslager et al., 2013). Research indicates that, on average, adolescents with a migration background remain disadvantaged in several ways, including in terms of educational outcomes (e.g. Baysu et al., 2021).

## The Current Study

Teacher ethnic/racial discrimination is generally harmful for adolescents’ development, but not all adolescents are affected by it to the same degree. Previous research has indicated that their heritage identification can be a protective factor, yet inconsistency remains. This may depend on adolescents’ national identification, but in many states, like Belgium, they have regional identities as well. The present study seeks to make a unique contribution to the literature by examining how regional, national and heritage identities intersect, and thus affect the well-being of adolescents at risk for teacher discrimination. The following hypotheses will be investigated: students who experience ethnic/racial teacher discrimination will have more depressive feelings than students who do not experience teacher discrimination (hypothesis 1); adolescents who are (nationally, not regionally) integrated will have lower depressive feelings than students from students with other combinations of identifications (hypothesis 2); for assimilated and separated adolescents, depressive feelings will not be higher when they perceive teacher discrimination (hypothesis 3).

## Methods

### Participants and Procedure

For this study a sample of 439 Turkish and Moroccan students is used (*M*_*age*_ = 18, *SD* = 0.92) participating in the Racism and Discrimination in Secondary Schools Survey (RaDiSS II, see Vervaet, 2018). Boys and girls were equally represented in the sample, with the reference group (boys) at 51%. Students were also asked to indicate in which track they were enrolled: academic, technical or vocational track. The students in the academic track (13%) functioned as reference group for the two dummy variables from the technical track (31.7%) and vocational track (55.4%). The socio economic status (SES) of a student was measured using the International Socio-Economic Index of Occupational Status (ISEI) (Ganzeboom et al., 1992). This metric variable was measured on the basis of the profession of the student’s parents. The highest ranked occupation of the parents was used to determine a final score assigned to every student. The higher the score, the higher the SES of a student, with the range being from 16 to 90 (*M* = 37.45, *SD* = 12.36). The majority of the Turkish and Moroccan students were second generation migrants (62.1%), compared to 17.6% first generation and 20.3% third generation migrants (missing = 0.2%).

The data was collected during the school year of 2014–2015 and contains information from 45 high schools in Flanders. The response rate of the schools was 53%. Due to the multistage sampling frame, this sample was ideal for studying interethnic relationships. In a first stage multicultural Flemish districts were selected. In the second stage, all the schools in these districts were divided into three categories according to the proportion of ethnic minority students. From each of these three categories, schools were then randomly sampled. Of the 45 schools involved, one-third had a low proportion (<15%, *M* = 5%, *SD* = 4; *M*_*ses*_ = 58, *SD* = 6.12) of ethnic minority students, one-third had a medium proportion (15–49,9%, *M* = 35%, *SD* = 17; *M*_*ses*_ = 48, *SD* = 5.77) and one-third had a high proportion (>50%, *M* = 72%, *SD* = 14; *M*_*ses*_ = 40, SD = 4.79) (Vervaet, 2018). The majority of the schools were situated in an urban to suburban environment. The students who filled in the survey were in the last year of secondary education (the 6^th^ year (*M*_*age*_ = 18), or similar to the 12^th^ grade in the US educational system). All students were older than 16 year of age and therefore parents were given the opportunity to give passive assent. Students participated voluntarily after being informed about the research goals and anonymous data processing. Students gave their full consent. This procedure is in line with Belgian ethical requirements for academic research. The response rate was 82%. The main reason students did not participate was because they were absent on the day of the survey. The paper-and-pencil survey was filled in by the students under the supervision of a researcher and one school staff member. Initially, the surveys were not anonymous. The names of the students were removed permanently once the data was entered in the computer.

### Measures

#### Depressive feelings

Students’ depressive feelings were assessed with a self-report scale (i.e. the CESDR-10, Haroz et al., 2014). Students were asked to indicate for ten items, on a 5-point scale (ranging from 1 – “never” to 5 –“very often”), how often they experienced poor appetite, restless sleep, sadness, disinterestedness in daily activities, tiredness, joy, happiness etc. Haroz et al. (2014) found strong psychometric properties for this shortened scale for two general samples. However, because this study was conducted on a very specific sample of adolescents from Turkish and Moroccan origin who all identified as Muslims, it was checked whether all these ten items could be included in one scale, specially the item related to suicidal thoughts. Suicide is an absolute taboo and unforgivable sin within Islam; it is known that through surveys and therapeutic interviews this sensitive topic should not be directly discussed (Walpole et al., 2013). First, to determine the number of factors Horn’s parallel analysis (Horn, 1965) was applied to all 10 items (for the application in SPSS see O’Connor, 2000). To determine the right amount of factors, the eigenvalue of the actual data should be higher than the eigenvalue of the randomly generated data. The results of this analysis showed that the items are underpinned by one factor structure. The principal component analysis (*KMO* = 0, 868) is then recalculated with the correct number of factors (çokluk & Koçak, 2016). The ten factors of the first factor explain 41% of the variance. The factor loadings ranged from 0.207 to 0.751. These items were averaged into a scale (*M* = 2.25, *SD* = 0.704, *α* = 0.829, *missing* = 0.7%, see Table 1 for descriptive statistics); the higher a student’s score, the more the student experienced depressive symptoms.

**Table 1:** Descriptive statistics—sample

Measures	Statistic	Turkish origin adolescents	Moroccan origin adolescents	Total sample
Ethnicity	%	41	59	100
Depression	Mean (SD)	2.250 (0.698)	2.244 (0.709)	2.247 (0.704)
Heritage belonging item 1	Mode	5	5	5
Heritage belonging item 2	Mode	5	5	5
Heritage belonging item 3	Mode	5	5	5
Belgian belonging	Mode	8	7	7
Regional belonging	Mode	8	7	7
Socio-Economic Status	Mean (SD)	37.37 (11.062)	37.51 (12.334)	37.45 (11.816)
Ethnic discrimination	%	35.0	31.7	67
No ethnic discrimination	%	65.0	68.3	33
Academic track	%	7.8	16.6	13
Technical track	%	38.3	27.0	31.7
Vocational track	%	53.9	56.4	55.4
First generation	%	13.4	20.5	17.6
Second generation	%	62.0	62.2	62.1
Third generation	%	24.6	17.4	20.3
Boys	%	60.0	55.2	51
Girls	%	40.0	44.8	49

#### Perceived teacher discrimination

To investigate perceived ethnic/racial teacher discrimination, every student was asked to indicate if they had ever been insulted, threatened, pushed, treated unfairly or excluded by teachers because of their foreign descent, language use, and skin colour (see also Pachter et al., 2010). Relying on the self-reports of adolescents is crucial in this matter as the interpretation of the situation is being discriminatory is part of phenomenon under study. The answer categories ranged from 0 to 6; zero representing “never” and six represented “all the time”. This frequency scale was highly zero-inflated and therefore discrimination was operationalized as a binary variable (*missing* = 0.9%). A sensitivity analysis was conducted with teacher discrimination as a metric variable. This analysis yielded the same results as the analysis presented in the result section. If students reported having experienced these victimizations, they were considered to have experienced ethnic/racial discrimination (33%), all the other students (67%) were considered not to have experienced ethnic/racial discrimination.

#### Identification

To map students’ multiple identification—three questions could be used from the dataset/survey. First, for the measurement of students’ heritage identity, the survey included items inspired by the MMRI and MIBI-teen centrality dimension (Schottham et al., 2008). Students had to respond to these items thinking about the nationality that first came to their mind and write that nationality down; for this study all the students who filled in Turkey and Moroccan were included. The three items included in the survey were: 1) when I introduce myself, I would definitely say I belong to this group, 2) I have a strong sense of belonging to this group; 3) and I see myself as a member of this group (based on Sellers et al., 1998). Students indicated on a 5-point Likert scale whether they completely disagreed (“1”) or agreed (“5”) with the statements. Second, to measure students’ belonging to the Belgian and Flemish identity groups they had to respond to the question: ‘”Do you feel a member of these groups” ranging from 1 (not at all) to 10 (totally). Low scores on this scale (<5) indicate a low belonging while high scores (<5) indicate a stronger belonging. Similar questions have been used to assess group membership (e.g. the “feeling thermometer”, Verkuyten & Thijs, 2010). All 5 identity measures were non-normally distributed and treated as ordinal variables with high values indicating a higher belonging and lower values indicating a lower belonging.

### Statistical Procedure

The analysis of this study was conducted in two steps. First, a Latent Class Analysis is conducted to identify participants’ multiple identity configurations. LCA is a statistical technique that aims at identifying latent groups within data based on categorical variables (Nylund-Gibson & Choi, 2018). Compared to variable-centered methods, person-centered methods—e.g. LCA—offer a parsimonious and readily interpretable way to capture heterogeneity in a multi-dimensional population. The advantage of this approach is its accommodation of intersectionality as an analytical tool (i.e. categories such as ethnicity, nationality etc. are interrelated and mutually shaping each other) (Collins & Bilge, 2020, p.2). The tidySEM package in R (Van Lissa 2019) is used to estimate the latent class solution. Several indicators have to be taken into account to select the number of classes. The optimal number of latent classes (i.e. identity configurations) is assessed by using the BIC and the AIC (Fraley & Raftery, 1998); with lower values indicating a better statistical fit. Solutions were considered to be admissible if they consisted of at least 5% of the sample (Depaoli, 2013), if the entropy (a measure of class separability) exceeded .8 (Wang et al., 2017), and if the solution was theoretically interpretable and qualitatively distinct from the other class solutions (Howard & Hoffman, 2018). For subsequent analyses, the most likely class membership based on posterior class probability was treated as an observed categorical variable whereby each category represented a different multiple identification strategy. These categories were interpreted by focusing on the mode of each identification measure within the different identification strategies. The mode corresponds to the ordinal measurement level of the identity measures. High belonging within a cluster is a mode higher than the midpoint of the ordinal scale, which is >3 for the items of heritage identification and >5 for national and regional identification. Low belonging is a mode lower than the midpoint of the ordinal scale (<3 for heritage identification and <5 for national and regional identification. A mode on the midpoint of the scale indicates a rather neutral stance towards that identification. This categorical variable is used for follow-up analysis in the second part of the result section.

Second, in order to investigate the moderating role of the multiple identification classes with respect to the link between perceived teacher discrimination and students’ depressive feelings, a hierarchical regression model from the software package HLM was estimated (i.e. students [level 1] grouped in schools [level 2])(version 8) (Raudenbush et al., 2013). Missing data was removed list wise by the software while running the analysis. The focus of this study is on individual level determinants and therefore no school level variables were entered in the analysis. Multilevel modelling is preferred considering the nested structure of the data. The regression was build up stepwise. All models are random-intercept models. No random slopes were tested. As a first step, an intercept-only model was estimated, to check if the intercept variance at the second level was significant. This indicated if multilevel analysis is necessary. Afterwards (model 1), the control variables were added (gender, track, SES). In model 2, hypothesis 1 is investigated which states that teacher discrimination will be associated with higher depressive feelings. Model 3 allows to check whether (nationally, not regionally) integrated adolescents have the most promotive identification in relation to depressive feelings (hypothesis 2). In model 4 interaction terms are added to examine whether multiple identifications moderates the relationships between teacher discrimination and depressive feelings by focusing on the main effect of teacher discrimination for each of these groups separately. This allows to investigate whether separation and assimilation function as protective factors (hypothesis 3).

## Results

### Adolescents’ Multiple Identification

The BIC indicated a two-class model as the best fitting solution (see Table 2), but a three class model was preferred because one of the classes in the two class solution was bimodal and therefore conceptually heterogeneous. Withholding theoretical and qualitatively distinct class solution is also an important indicator in model selection. The three class model made a the theoretically important distinction between adolescents’ regional and national identification as distinct from heritage identification. Both AIC and BIC still indicate that three classes are a good fit for the data. The BIC of the three class model was 59.25 points higher than that for the two-class solution. Both solutions were considered admissible (*entropy* = 0.9; *N min* ≥ 24%). It is important to take into account all these criteria (i.e. model fit, theoretical interpretability, entropy, minimum class size) when selecting cluster solutions (Tables 2 and 3).

**Table 2:** Turkish and moroccan students—model-fit indices—latent class analysis

N° profiles	P	AIC	BIC	Entropy	N min.
1	30	6203.16	6325.69	1.00	1.00
2	61	5743.07	5992.22	0.90	0.36
**3**	**92**	**5675.69**	**6051.47**	**0.90**	**0.24**
4	123	5641.97	6144.37	0.92	0.07
5	154	5574.83	6203.84	0.94	0.07
6	185	5588.91	6344.54	0.90	0.04

*N* = 439, *P* = number of parameters estimated, *AIC* Akaike Information Criterion, *BIC* Bayesian Information Criterion, *N min*. = smallest class proportion, bold row indicates the model withheld for further analysis.

**Table 3:** Turkish and moroccan students—results of the LCA 3 class solution—mode

		Profiles Turkish and Moroccan students		
		1	2	3
Variables		Integration	National Integration	Separation
	N class	155	177	107
	Overall sample mode	Within-profile mode		
Ethnic heritage identity belonging—item 1	5	4	5	5
Ethnic heritage identity belonging—item 2	5	4	5	5
Ethnic heritage identity belonging—item 3	5	4	5	5
National identity belonging	7	7	8	5
Regional identity belonging	7	7	1	5

*n* = 439

Adolescents in the first class (“Integration”) had generally a high belonging to their ethnic heritage identity and also a high belonging to both their national and regional identity. Interestingly, adolescents in the second class (“National integration”) had a high belonging to their ethnic heritage identity and national identity, but a considerable proportion of adolescents in this class had a low belonging to the regional identity. The second class was internally heterogeneous in terms of belonging to the regional identity. Lastly, adolescents in the third class (“[weak] Separation”) nearly unanimously indicated the strongest belonging to their ethnic heritage identity, while they generally had rather “neutral” feelings towards their national and regional identity.

### Teacher Discrimination and Adolescents’ Depressive Feelings: The Role of Multiple Identification

At the start of the multilevel modelling, the null-model is evaluated to check the extent of dependency in the data due to the nested structure (i.e. students within schools) (Hox et al., 2017). The intra-class coefficient (ICC) showed that 3.0 percent of the variance in depressive feeling was situated at the school level (level 2)(*p* = 0.056). Due to the nested structure of the data, multilevel modelling was preferred. The results of the multilevel analysis are presented in Table 4.

**Table 4:** Random intercept multilevel analysis on students’ depressive feelings—reference groups: national integration & perceived teacher ER discrimination

Variables		Depressive feelings		
	Model 1	Model 2	Model 3	Model 4
Intercept	2.476*** (0.120)	2.568*** (0.124)	2.413*** (0.131)	2.352*** (0.139)
**Student track**: (*ref. academic trac*k)				
Vocational track	−0.344*** (0.103)	−0.323** (0.103)	−0.274** (0.101)	−0.270** (0.101)
Technical track	−0.143 (0.109)	−0.146 (0.109)	−0.088 (0.107)	−0.089 (0.107)
**Student sex**: (*ref. boy*)				
Girl	0.130 (0.067)	0.173** (0.068)	0.196** (0.067)	0.193** (0.067)
**SES**	−0.001 (0.003)	−0.002 (0.003)	−0.001 (0.003)	−0.001 (0.003)
**Student migrant generation**: (*ref. Third generation/native)*				
First generation	−0.090 (0.109)	−0.072 (0.108)	−0.080 (0.107)	−0.075 (0.107)
Second generation	−0.073 (0.084)	−0.072 (0.084)	−0.062 (0.082)	−0.060 (0.083)
**Teacher discrimination**: (*ref. ER discrimination)*				
No ER discrimination		−0.191** (0.072)	−0.222** (0.072)	−0.132_(a)_ (0.108)
**Student multiple identification**: (*ref. national integration*)				
Separation			0.066 (0.083)	0.224 (0.135)
Integration			0.273*** (0.075)	0.322* (0.135)
**Interaction terms**:				
Separation × No ER discrimination				−0.252_(b)_ (0.171)
Integration × No ER discrimination				−0.080 (0.162)
Deviance	904.587***	896.853**	883.472***	881.276

Standard Errors between brackets. No school level/level 2 variables were included in the analysis. No random slopes were tested. Significance levels: ***p < 0.001; **p < 0.01; *p < 0.05. In Table 4: teacher ER discrimination + national integration as reference group. Other relevant coefficients (Model 4*, ∆*_*a-b*_) reported in the text

Model 1 – which included the control variables – showed that students in the vocational track experienced less depressive feelings than students in the academic track. Female students experienced more depressive feelings than male students. There was no significant relationship between adolescents’ depressive feelings and socio-economic status nor adolescents’ migration history. Model 2 showed that students who perceived no teacher discrimination had less depressive feelings than students who perceived teacher discrimination (reference group), this confirms hypothesis 1. In model 3 adolescents’ identification classes were added. Students in the integration class had significantly higher depressive feelings than students in the national integration class. Students in the separation class also had more depressive feelings than students in the national integration class but this difference was not significant (reference group). Therefore hypothesis 2 is partially confirmed that national integration is positively associated with adolescents’ mental health. The significant difference in depressive feelings between fully integrated and national integrated students showed that high or low belonging to the regional identification was important. In model 4 interactions were added to understand the moderating role of multiple identification in case of perceived teacher discrimination (*χ*^*2*^ (2, *n* = 439) = 2.66, *p* = 0.368). Adding the interactions does not improve the model fit and therefore there is not sufficient evidence that multiple identifications moderated the relationship between perceived teacher discrimination and depressive feelings. Therefore hypothesis 3 cannot be confirmed, that proposed separation is a protective factor for the negative impact of discrimination. Swapping the reference group and analyzing the slope for ethnic discrimination indicated that this effect might potentially only be significant for students in the separated class (see Table 4, model 4, ∆_a b_ [*b*_*separation*_ = −0.382, *SE* = 0.102, *p* < 0.001]), however the evidence for this result is rather weak.

## Discussion

Previous research studied the role of adolescents’ heritage identity as a buffer between discrimination and mental health (Umaña-Taylor, 2016) and more recently, scholars adopted a bi-dimensional approach, based on the acculturation framework (Berry, 1997), incorporating adolescents’ heritage and national identity. However, in many European societies, adolescents with a migration background also develop a regional identity, indicating that a bi-dimensional approach may not suffice. This study investigated how adolescents from Turkish and Moroccan background combined these three identities and how they related to depressive feelings generally and/or as a buffer for the experience of teacher discrimination. In general the results showed that discrimination leads to higher depressive feelings and that taking into account the national-regional distinction is relevant to understand the well-being of adolescents with a Turkish and Moroccan migration background.

More broadly, three multiple identification classes were discovered. Thirty-five percent of all the students in the sample could be classified as fully integrated: a multiple identification in which high ethnic heritage identification is combined with high national and high regional identification. Previous research (e.g. Berry & Hou, 2017) found that integration is the most popular/common acculturation strategy but this is not the case in this sample. The most common multiple identification that was discovered was national integration (40%); adolescents in this group mostly had very low regional belonging although there was considerable internal heterogeneity. Twenty-four percent of the adolescents in this study had a (weakly) separated identification (i.e. strong belonging to the heritage identity and moderately positive/neutral identification with the (sub) national identity). The latter two classes show that for many adolescents multiple identification takes on a clearly divided structure and thus suggest that having multiple in-groups can be challenging. Interestingly, the marginalization and assimilation classes that have been theoretically described did not inductively emerge from this data. For all adolescents, their heritage identification was very important.

These distinct multiple identification classes also related differently to adolescents’ depressive feelings. Previous research indicated that integration is generally promotive for adolescents’ mental health (Schotte et al., 2018) and that combining national and regional identities can be a struggle for adolescents’ with a migration background (Driezen et al., 2021). It was therefore hypothesized that nationally integrated adolescents would report less depressive feelings than students with other multiple identifications (hypothesis 2). This hypothesis can partially be confirmed. Weakly separated and fully integrated students reported more depressive feelings than nationally integrated students, but only for the latter were these significantly higher. Adolescents in the fully integrated class were potentially more sensitive to the civic nationalist—ethnic sub nationalist political discourse within Belgium (e.g. Leong et al., 2020) and might therefore experience “identity-stress” as a confirmation of this duality that is present within Belgian society. This nuance in ethnic or civic discourses can be interesting for identity scholars who focus on other (sub) nations within Europe such as Catalonia (e.g. Ruiz Casado, 2019) and want to understand the repercussions of these identity politics for people’ daily lives (e.g., their mental health).

Previous research suggested that assimilation (Berry & Hou, 2017) and separation (Baysu et al., 2011) are protective factors for adolescents’ experience of teacher discrimination (i.e. hypothesis 3). It was not supported by the analysis in this study. If anything, the relation between teacher discrimination and depressive feelings was stronger for segregated students, but this effect was not statistically significant. This is somewhat in line with the finding by Berry and Hou (2017) that separated students who perceive ethnic/racial discrimination have a lower life satisfaction. Separated/segregated students might experience discrimination more intense, for example because of their strong connection to their heritage, while simultaneously lacking a bond with the social groups that discriminates them. While separation seems to have negative psychological consequences in the case of discrimination, this was not the case for academic achievement in another study which found it to be a protective factor in high threat situations (Baysu et al., 2011). This suggests that multiple identification might function differently according to the outcomes of interest and should be studied in more detail.

Two other relevant findings emerged from the analysis concerning gender and tracking. This study confirms that females have higher depressive feelings than male adolescents (Rueger et al., 2016; McLaughlin & King, 2015). The analysis also showed that students enrolled in the vocational track had less depressive feelings than students in the academic track. This corroborates with other international research (Yi et al., 2012) that discovered that high competitive environments that are structurally embedded in the educational system can cause high levels of depression for students in the academic track. This finding deserves more attention in future research on educational inequality and adolescents’ development.

### Implications

These results are also of importance to educational policy makers, educational boards, and teachers more generally. This study adds to a long list of empirical evidence that perceived ethnic/racial teacher discrimination is harmful for adolescents’ development. Educational actors can be aware that it is in fact enough for an adolescent to view a situation as discriminatory to be directly linked to that person’s well-being. Initiatives that promote students to talk from their point view and experiences could stimulate positive personal development and reflection in teachers on how they (un)consciously discriminate against students. Another interesting finding is that a lot of variation exists in how adolescents identify with important societal groups. In the Belgian context specifically, teacher are often aware that Turkish and Moroccan origin adolescents strongly identify with their countries of origin but tend to overlook how diverse that group of adolescents is in terms of their belonging to other meaningful identities such as the national and regional identification. In that regard, this research certainly shows that a strong attachment to one’s country of origin does not stand in the way of belonging to another nation and/or regions.

### Limitations

This study has several limitations. First, the design relied solely on self-reported measures. Although this is the only way to measure self-ascribed identification, it increases the risk of common method bias between predictors and the outcome. This also means that this study measured the extent to which adolescents *perceived* situations with their teachers to be ethnically or racially motivated, rather than a more objective measure. One advantage, of using self-report is that it reflects whether students interpreted the behavior as discriminatory. The analysis suggests that this perception is meaningfully associated with mental health for a considerable group of adolescents in the sample. In this study, ethnic discrimination was approached as a binary event and it was not possible to properly distinguish between two important dimensions of discrimination i.e. the frequency and the severity of those incidents. Second, this study is based on a cross-sectional design, which means that causality cannot be established based on these findings. Longitudinal research on discrimination, identification and mental health is necessary to fully interpret current findings. Third, this study applied sequentially two analysis techniques. First a LCA was conducted and afterwards these results were used to test hypotheses with multilevel regression modelling. A limitation of this approach is that potential classification error from the LCA is not accounted for in the multilevel analysis. However, the impact of this limitation is likely to be small, as the classes were highly distinct (*entropy* = .90; Celaux & Soromenho, 1996).

### Future Research

The present study adds to the literature by providing further evidence of the detrimental relationship between discrimination and depressive feelings. Future research could however fine tune knowledge on discrimination by focusing on the potentially differing role of micro-aggressions in respect to discrimination as a blunt incident. Micro-aggressions are more subtle (unintentional) forms of discrimination that happen during every day micro-interactions (Ogunyemi et al., 2020) and thus far the relationship between micro-aggressions and mental health is not well established (for an example, Nadal et al., 2014). Another strength of this study is the explicit focus on the importance of sub national / regional identification in combination with heritage and national identification and its association with students’ depressive feelings. This is an important step forward in measuring people’s identification more accurately. This study—together with other recent studies on multiple identification (e.g. Clycq et al., 2021—focusing on the importance of transnational identity; Maene et al., 2021—taken into four relevant identities among which religious identity and compares adolescents’ with and without migration background with each other; Verkuyten et al., 2019—theoretical contribution)—opens the door for other scholars to take into account other important social identities and to focus on their interplay and their relation to both social and psychological outcomes. It seems to us an important avenue for further research to possibly look at how different groups of adolescents (vis-à-vis their multiple identification) perceive and experience ethnic discrimination, especially in bigger samples, as research results are thus far inconsistent in their relationship between identification profiles and their outcomes. With this in mind, future research might also investigate which identities are relevant to adolescents with other ethnic backgrounds and how these intersect which each other. Qualitative interviews could further explore adolescents’ perceptions of these identities and quantitative research could also involve other aspects of adolescents’ identification such the civic versus ethnic representations of their national and regional identities. These are aspects of identity that are usually studied in samples that do not have a migration background. Yet, with many minority adolescents in secondary education being second or third generation migrants these other are (increasingly) relevant.

## Conclusion

Adolescents’ heritage identity has been proposed as a protective factor against ethnic/racial discrimination, but the literature has yielded inconsistent findings, which may be due to the intersections of heritage, national, and regional identities. This study investigated the link between perceived teacher discrimination and adolescents’ depressive feelings, and their multiple identification as potential source of resilience. The results show that adolescents’ multiple identities are highly distinct and that the difference between their national and regional identity is important in addition to their heritage identity. This also implies that adolescents who are entering adulthood already have taken position vis-à-vis the political/organization structure of the country in which they live and that this is affecting their well-being. The results indicate that adolescents who belong to both the nation and the region have more depressive feelings than adolescents who only belong to the nation. Moreover, adolescents’ experience of teacher discrimination was linked to higher depressive feelings and this seemed particularly strong for the group of adolescents with a separated identification. More research is needed as a separated identification may render these adolescents particularly vulnerable to discrimination-related mental health problems.
